# The identification and management of ADHD offenders within the criminal justice system: a consensus statement from the UK Adult ADHD Network and criminal justice agencies

**DOI:** 10.1186/1471-244X-11-32

**Published:** 2011-02-18

**Authors:** Susan J Young, Marios Adamou, Blanca Bolea, Gisli Gudjonsson, Ulrich Müller, Mark Pitts, Johannes Thome, Philip Asherson

**Affiliations:** 1 King's College London, Institute of Psychiatry, De Crespigny Park, London, SE5 8AF, UK; 2 South West Yorkshire Partnership NHS Foundation Trust, Manygates Clinic, Belle Isle Healthpark, Portobello Road, Wakefield, WF1 5PN, UK; 3 Psychopharmacology Unit, Dorothy Hodgkin Building, Whitson Street, Bristol, BS1 3NY, UK; 4 Department of Psychiatry, Box 189, Level E4, Addenbrooke's Hospital, Hills Road, Cambridge, CB2 0QQ, UK; 5 Adult ADHD Service, South London & Maudsley Trust, Maudsley Hospital, Denmark Hill, London, SE5 8AZ, UK; 6 Institute of Life Science, Swansea University, Singleton Park, Swansea, SA2 8PP, UK

## Abstract

The UK Adult ADHD Network (UKAAN) was founded by a group of mental health specialists who have experience delivering clinical services for adults with Attention Deficit Hyperactivity Disorder (ADHD) within the National Health Service (NHS). UKAAN aims to support mental health professionals in the development of services for adults with ADHD by the promotion of assessment and treatment protocols. One method of achieving these aims has been to sponsor conferences and workshops on adult ADHD.

This consensus statement is the result of a Forensic Meeting held in November 2009, attended by senior representatives of the Department of Health (DoH), Forensic Mental Health, Prison, Probation, Courts and Metropolitan Police services. The objectives of the meeting were to discuss ways of raising awareness about adult ADHD, and its recognition, assessment, treatment and management within these respective services. Whilst the document draws on the UK experience, with some adaptations it can be used as a template for similar local actions in other countries.

It was concluded that bringing together experts in adult ADHD and the Criminal Justice System (CJS) will be vital to raising awareness of the needs of ADHD offenders at every stage of the offender pathway. Joint working and commissioning within the CJS is needed to improve awareness and understanding of ADHD offenders to ensure that individuals are directed to appropriate care and rehabilitation. General Practitioners (GPs), whilst ideally placed for early intervention, should not be relied upon to provide this service as vulnerable offenders often have difficulty accessing primary care services. Moreover once this hurdle has been overcome and ADHD in offenders has been identified, a second challenge will be to provide treatment and ensure continuity of care. Future research must focus on proof of principle studies to demonstrate that identification and treatment confers health gain, safeguards individual's rights, improves engagement in offender rehabilitation programmes, reduces institutional behavioural disturbance and, ultimately, leads to crime reduction. In time this will provide better justice for both offenders and society.

## Introduction

UKAAN was established in 2009 in response to UK guidelines issued by the National Institute for Clinical Excellence (NICE) in 2009 [1] and the British Association of Psychopharmacology [2] which for the first time gave evidence based guidance on the need to diagnose and treat ADHD in both adults and children.

ADHD is a clinical syndrome defined in the Diagnostic and Statistical Manual - Fourth Edition (DSM-IV) and International Statistical Classification of Diseases - Tenth Revision (ICD-10) by high levels of hyperactive, impulsive and inattentive behaviours beginning in early childhood. The disorder is common in the population with prevalence estimates in the UK of around 3-4% [3]. Follow-up studies of ADHD in children find that the disorder frequently persists with around 15% retaining a full diagnosis by 25 years, and a further 50% retaining some symptoms leading to continued impairments in daily life [4]. A recent review and meta-analysis estimated the world prevalence in adults to average 2.5% or higher [5]; with around 1% expected to fall in the most severe group requiring immediate treatment. In the UK, the rate of adult ADHD has been estimated at 1% [3].

While ADHD-like symptoms are found in many people some of the time, in people with ADHD they are severe, persistent over time and lead to clinically significant impairments. Impairments can impact on an individual in several ways including: low self-esteem, educational and occupational problems, problems in social interactions and relationships, antisocial behaviour, the development of comorbid psychiatric symptoms, syndromes and disorders, and the capacity to cope with police interviews and court procedures [1]. Comorbidities in ADHD are common and include other neurodevelopmental disorders such as autism spectrum disorders and dyslexia, drug and alcohol abuse disorders, personality disorder, or other common mental health problems such as anxiety and depression [1].

### ADHD in Forensic Settings

Research suggests there is a disproportionately high concentration of ADHD individuals involved with the CJS, and for these individuals criminal justice procedures often interface with a complex web of behaviour, substance use and mental health issues. International studies from the USA [6], Canada [7], Sweden [8,9], Germany [10-12], Finland [13] and Norway [14] report that up to two-thirds of young offenders and half of the adult prison population screen positively for childhood ADHD, and many continue to be symptomatic with rates reported at 14% in adult male offenders [15] and 10% in adult female offenders [10]. In young offenders rates are around 45% [12,16]. A UK study of personality disorder wards in Forensic Mental Health Services found similar screening rates (33%), with a sizeable number of individuals in partial remission of symptoms [17].

UK prison studies have indicated a rate of 43% in 14-year-old youths [16] and 24% in male adults screening positive for a childhood history, 14% of whom had persisting symptoms [15]. Those with persisting symptoms accounted for eight times more aggressive incidents than other prisoners and six times more than prisoners with Antisocial Personality Disorder. They had a significantly younger onset of offending by around 2.5 years (16 vs. 19.5 years); and they had a significantly higher rate of recidivism [18]. ADHD was the most important predictor of violent offending, even above substance misuse.

Thus the rate of ADHD in the CJS far exceeds that in the general population, and offender behaviour, both within and outside of prison settings, is something that society cannot afford to ignore. The higher rate of ADHD individuals involved in the CJS however is not paralleled by the knowledge, skills and training of practitioners in the disorder and who are involved in their care. NICE Guidelines for ADHD [1] were comprehensive in their recommendations for service delivery, emphasising the need for integrated services reflecting developmental needs across the lifespan, including forensic services. Establishing who, out of the 'mixed bag' of individuals within forensic services has a diagnosis of ADHD and will benefit from ADHD treatment as a first-line primary intervention (rather than treatment targeting substance use or other mental health problems) is an important question.

### Offender Health

In the past few years commissioning responsibilities for prison healthcare have transferred from the prison service to the NHS in order to:

1) Increase investment in prisoner health.

2) Raise services to NHS standard.

3) Provide continuity for those in prison who later return to communities.

A central tenet is that prisoners should be considered as part of the community and treated within mainstream services with access to the same standards of health and social care as the rest of the population. Nevertheless, it is recognised that there exists a sub-group of individuals who have particular difficulty navigating the system, perhaps due to poor educational ability, disturbed mental state, and/or substance misuse. By supporting these individuals in their care and through the provision of integrated services, the justice they receive will also be supported.

Offender Health now exists as a partnership between the Ministry of Justice and the DoH and, to date, the focus has been to:

1) Develop mental health transfer protocols to facilitate the transfer of those with severe mental illness to mental health settings.

2) Introduce an Integrated Treatment System, which draws together clinical interventions for prisoners (e.g. methadone maintenance and psychosocial interventions).

In response to the recommendations of the Bradley Report [19], a national Health and Criminal Justice Programme Board has been set up, bringing together government departments for health, social care and criminal justice. The Board have devised a National Delivery Plan [20] committed to improving the management of offenders with mental health problems, learning disability and personality disorder, which provides an opportunity to move ADHD up the care agenda. Its key objectives are to:

1) Improve system effectiveness and efficiency.

2) Work in partnership.

3) Improve capacity and capability.

4) Develop an equity of access to existing general services and/or specialised services for ADHD.

5) Improve pathways and continuity of care.

The Health and Criminal Justice Programme Board is supported by a National Advisory Group, which provides independent, evidence-based advice to the Board on the developing agenda, and highlights examples of good practice and the commissioning of in-depth studies in areas of interest. Thus this National Advisory Group will provide a mechanism for UKAAN to raise the profile of ADHD offenders at the highest level.

However, the volume and scale of activity within the CJS will influence what can realistically be achieved in terms of ADHD screening, assessment and treatment, and new developments must be integrated with existing protocols and run in a system at high capacity. Health inequality is common in the prison population for many reasons (e.g. personal and socio-economic, community, lack of continuity, failure to access general services), and the DoH has expressed commitment to raising standards for the benefit of prisoners and with a view to improving longer term outcomes such as a reduction in reoffending and positive integration into the community. However there is no 'quick fix' as most prison inmates are young men with complex healthcare needs, including alcohol and substance misuse problems and psychological problems. On the other hand health assessments and interventions often have to be rapidly implemented as approximately half of prison inmates stay in prison for an average of six months or less. Nevertheless there is room for innovation - screening at prison reception has improved and non-health staff are now involved in a preliminary screening process, which triggers a more comprehensive assessment, if required, conducted by health staff. The Integrated Treatment System is the appropriate pathway for introducing ADHD assessment and management as this will include after-care arrangements, e.g. for treatment post-discharge.

### The Bradley Report

The Bradley Report [19] was commissioned in December 2007 to examine the extent to which offenders with mental health problems or learning disabilities could, in appropriate cases, be diverted from prison to other services and the barriers to such diversion; and to make recommendations to government, in particular on the organisation of effective court liaison and diversion arrangements and the services needed to support them. The focus was expanded to include a more comprehensive consideration of the 'offender pathway' and associated mental health services, and in compiling the report Lord Keith Bradley visited a wide range of facilities throughout the country. Nationally, Lord Bradley's Report makes over 80 recommendations to Government which would ensure public protection, appropriate justice and that people with mental health problems or learning difficulties are identified and treated as they pass through the CJS and re-enter society. The Bradley Report predominantly focused on adults with mental health problems and learning disability, and ADHD does not fit well within either category. Nevertheless, the Bradley Report has some translational value for youths and adults with ADHD. Thus these proceedings highlight key recommendations of the Bradley Report where deemed appropriate. Table 1 presents key recommendations across criminal justice services and Table 2 presents key recommendations for youth services from the Bradley Report Executive Summary [19].

**Table 1:** Key recommendations made in Bradley Report (2009) across criminal justice services

- Improve awareness, identification, assessment and training in mental health needs.
- Ensure qualified individuals exist within services to make appropriate referrals.
- Review the potential for early examination and intervention in childhood.
- Form closer links between services (e.g. joint-training packages, information sharing).

**Table 2:** Key youth recommendations from the Bradley Report Executive Summary (2009)

- Youth Offending Teams must include a suitably qualified mental health worker who is responsible for making appropriate referrals to services.
- The Government should undertake a review to examine the potential for early intervention and diversion for children and young people with mental health problems or learning disabilities who have offended or are at risk of offending, with the aim of bringing forward appropriate recommendations which are consistent with this wider review.

## Identification and Screening Procedures

Currently the National Criminal Justice Board meets regionally and nationally, with representation by the courts, police, probation and prison services. Screening systems already exist in CJS services and we need to identify ways of building on these systems to incorporate screening for ADHD. Making representations to the National Criminal Justice Board might be one way to move forward. In developing an effective and efficient screening protocol for ADHD within various CJS settings and in developing appropriate care pathways, it will be important to determine the level of awareness that exists in services, what screens are currently used, and what a positive screen triggers in terms of individuals progressing through CJS procedures and services.

### Police Services

Table 3 presents recommendations for policing and community care from the Bradley Report Executive Summary [19]. The culture of present day policing is heading towards a crime reduction strategy, and new procedures and performance indicators have been introduced in order to maximise crime reduction and improve cost-efficiency. However, busy police custody suites manage a high turnover of detainees (more than half of whom are intoxicated), which complicates any systematic screening. The Police and Criminal Evidence Act led to improved recording of information and data are now recorded about an individual's behaviour, physical and mental health. However mental health needs are not perceived to be a priority. Thus internal cultural changes will be required to raise awareness and recognition of ADHD. Training opportunities are available for police officers, in particular for custody officers who complete initial and refresher training in line with new legislation or developments.

**Table 3:** Recommendations for policing and community care from the Bradley Report Executive Summary (2009)

- Local Safer Neighbourhood Teams should play a key role in identifying and supporting people in the community with mental health problems or learning disabilities who may be involved in low-level offending or anti-social behaviour by establishing local contacts and partnerships and developing referral pathways.
- Community support officers and police officers should link with local mental health services to develop joint training packages for mental health awareness and learning disability issues.
- A review of the role of Appropriate Adults in police stations should be undertaken and aim to improve the consistency, availability and expertise of this role.
- Appropriate Adults should receive training to ensure the most effective support for individuals with mental health problems or learning disabilities.
- Mental health awareness and learning disabilities should be a key component in the police training programme.
- All police custody suites should have access to liaison and diversion services. These services would include improved screening and identification of individuals with mental health problems or learning disabilities, providing information to police and prosecutors to facilitate the earliest possible diversion of offenders with mental disorders from the criminal justice system, and signposting to local health and social care services as appropriate.
- Liaison and diversion services should also provide information and advice services to all relevant staff including solicitors and Appropriate Adults.

The Criminal Intelligence System database includes mental health data and, once improved, screens will be standardised and introduced nationally providing an efficient and cost-effective way of sharing data and alerting staff to particular needs (in line with confidentiality legislation). Currently a Risk Assessment screen is given to every person received into custody and this includes questions about current mental state (e.g. risks posed by depression, suicidal ideation and self-harm). This triggers a follow-up primary care screen within 48 hours (to which ADHD items could be added) and/or contact with a forensic medical examiner to ensure that the individual is fit to be detained and interviewed. It also identifies individuals who require regular observations (e.g. to prevent suicide). For those fit for interview, other provisions can be made. In the UK for example, if a detainee is suspected of having a mental health need they must be supported by an appropriate adult (AA) during interview. The AA can give advice to all parties, furthers communication and ensures that the interview is fair, however even when ADHD is recognised, detainees will not necessarily be entitled to an AA unless triggered by some additional problem (e.g. learning disability). It is important to note that for many young offenders the AA will be a parent and, given the hereditary nature of ADHD, this in itself may have implications for the custody process. Furthermore, some countries do not have the AA system in place, in which case the vulnerability of detainees with ADHD (recognised or unrecognised) is more serious as they get no additional support. It is recognised that the introduction of improved screening may result in more detainees requiring an AA, and a revised AA scheme is due to be introduced, providing opportunities to introduce ADHD training and/or psychoeducational materials on ADHD recognition, treatment and management.

In completing any screen, detainees may be resistant to engage with officers who have arrested or detained them, thus it is important that screens are completed sensitively to avoid disclosure being limited if detainees perceive stigma associated with their endorsing mental health problems. Language barriers are routinely overcome by the use of interpreters who can attend the police station within two hours. Cultural barriers also need consideration, as does the perception that if a mental health need is disclosed or suspected, the criminal justice process will be lengthened.

### Courts Services

The need for close working relationships between health professionals and the courts has been documented in The Bradley report [19] (see Table 4) and by the DoH [21,22] and a merging of services is clearly taking place [23]. Her Majesty's Courts Service has responded to the Bradley recommendations by considering the implementation of Criminal Justice Mental Health Teams, and the first specific courts for offenders with mental health problems or learning disabilities have been piloted in Brighton and at Stratford magistrates' courts. Nevertheless, it is recognised that provision of diversion schemes varies throughout the country with some areas relying on the voluntary sector and some having no support at all [24], while others have designated workers providing forensic support to youths and adults. Both the Magistrates and Crown Court Judiciary receive training provided by the Judicial Studies Board. The Magistracy has a Bench Book specifically concentrating upon equal treatment which has some details of ADHD, but it is not known how widely this is utilized within the courts. Specific training in mental health is not provided for Magistrates but it is available for Crown Court Judges.

**Table 4:** Key recommendations for Court and Probation Services from the Bradley Report Executive Summary (2009)

- Information on an individual's mental health or learning disability needs should be obtained prior to an Anti-Social Behaviour Order or Penalty Notice for Disorder being issued, or for the pre-sentence report if these penalties are breached.
- The Crown Prosecution Service should review the use of conditional cautions for individuals with mental health problems or learning disabilities and issue guidance to advise relevant agencies.
- Immediate consideration should be given to extending to vulnerable defendants the provisions currently available to vulnerable witnesses.
- Courts, health services, the Probation Service and the Crown Prosecution Service should work together to agree a local service level agreement for the provision of psychiatric reports and advice to the courts.
- The judiciary should undertake mental health and learning disability awareness training.
- Liaison and diversion services should form close links with the judiciary to ensure that they have adequate information about the mental health and learning disabilities of defendants, and concerning local health and learning disability services.
- All probation staff (including those based within courts and approved premises) should receive mental health and learning disability awareness training.
- Further work should be undertaken to ensure better implementation of the Care Programme Approach for people with mental health problems in prisons, to ensure continuity of treatment through the prison gate.

Should ADHD be recognised at any stage of the court process, it could be referred as necessary to health professionals and/or the Probation Service to assist the court in its sentencing decisions. The National Probation Service provides pre-sentence reports to assist the judiciary with sentencing decisions. Some reports are described as 'Standard Delivery' taking up to three weeks (i.e. involving more serious offending and/or complexity of offender needs) and others are 'Fast Delivery' taking up to five days. Considerations necessitating the request of psychiatric reports arise from Section 157 of the Criminal Justice Act 2003 which places an obligation upon the court to consider a medical report in "any case where the offender is or appears to be mentally disordered" (s157 (1)) "unless the court is of the opinion it is unnecessary" (s157 (2)). Section 207 of the same Act also requires evidence of a registered medical practitioner if a mental health treatment requirement as part of a community order is required. Currently some court areas are developing service level agreements for the provision of such reports as suggested within the Bradley Report [19].

There is a Government expectation that the proportion of Fast Delivery Reports will increase to 70%, therefore ADHD screening needs to be built into initial screening processes (which vary across probation areas) in order to flag up whether the greater level of assessment provided by a Standard Delivery Report is required. With this in mind, probation staff would need training to screen for ADHD and learn how and from where to access diagnosis and treatment. The most likely procedure would be referral to a forensic psychiatric service for a comprehensive assessment. An area for development is for Local Criminal Justice Boards to establish effective protocols with health service providers to ensure that there are cost effective and practical arrangements for diversion and treatment for Court users with mental health problems and/or learning disabilities.

### Probation Services

As part of the National Offender Management Service, the probation service is made up of 42 Probation Trusts that operate independently from each other to manage offenders and monitor them through the orders imposed by the courts (Sentences). Offender Managers provide interventions, (e.g. Accredited Programmes, Employment Training and Education and Community Payback) and monitor their clients' progress and, while there are national standards, each Trust and is encouraged to tailor responses to local needs and priorities and the offender profiles within their areas. Joint Needs Assessments are thus conducted between the National Offender Management Service and Primary Care Trusts (PCTs) resulting in targeted Offender Care Pathways that also reflect national initiatives (this is captured within the regional Offender Health Delivery Plan). One such initiative is the provision of mentoring/peer education services invested in by Probation Trusts and PCTs (e.g. the emergence of 'Peer Health Educators'). These initiatives are in the early stages of development (relatively speaking) but have a significant role to play in an offenders' journey as they provide continual support for the offender from custody to the community. Thus Peer Health Educators could develop their knowledge and skills about ADHD and prompt referrals from Offender Managers.

A useful tool for the identification of need is the Offender Assessment System (OASys), which has the potential to provide further determination of what barriers may exist for an offenders' ability to adhere to their rehabilitation requirements. OASys provides an opportunity for the identification of non-criminogenic needs with work ongoing to identify how Offender Managers can be made aware of issues such as ADHD, thus influencing the care pathway for an individual, and the use of OASys for this purpose could well be in addition to any local assessment tools that exist.

Whichever stage an offender is at (police, courts, prison, on licence) a protocol would need to be established for the effective identification of which offenders have ADHD so that this can be taken into account in terms of assessing offending behaviour (e.g. court reports, proposals made to sentencers) and ensuring that the interventions meet offender needs (in order to maximise their chances of compliance and successful completion). Any protocol would need to be established with each Probation Trust, ideally working in partnership with other agencies, including health, thus providing the best means of ensuring that the needs of offenders with ADHD are identified, diagnosed and met.

### Prison Services

Table 5 presents key recommendations for the prison service from the Bradley Report Executive Summary [19]. In most areas PCTs are responsible for contracting for prison health care at a primary level (i.e. GPs provide primary medical input and go into prisons on a sessional basis) and at a secondary level (usually provided by an adjacent Trust). Thus commissioners could request that ADHD screening, assessments and interventions are included under this care contract. There are several opportunities within the prison care system through which ADHD could be identified:

**Table 5:** Key recommendations for the prison service from the Bradley Report Executive Summary (2009)

- A study should be commissioned to consider the relationship between imprisonment for public protection sentences and mental health or learning disability issues.
- An evaluation of the current prison health screen should be undertaken in order to improve the identification of mental health problems at reception into prison.
- NHS commissioners should seek to improve the provision of mental health primary care services in prison.
- Prison mental health teams must link with liaison and diversion services to ensure that planning for continuity of care is in place prior to a prisoner's release, under the Care Programme Approach.
- Awareness training on mental health and learning disabilities must be made available for all prison officers.

1) Primary care health workers.

2) Mental health in-reach teams.

3) General forensic psychiatrists.

4) GPs.

5) Specialist learning disability nurses.

More informally, wing staff are the 'eyes and ears' of the prison. They interact with inmates on an intensive, daily basis and, whilst they usually lack the ability to describe perceived difficulties in medical terms, they are well placed to identify when a prisoner is 'different' or unwell.

Prison reception health screens are currently being reviewed. The current procedure (the 'Grubin' screen) is a two-part procedure comprising a brief screen for depression and suicidal ideation followed by a more comprehensive health screen to which ADHD items could be added. Currently around half of individuals entering the prison system complete both sections. While ADHD could be incorporated into this screen, it is important to maintain the brevity of the screen. Furthermore, several needs will compete with ADHD for inclusion (e.g. autism, learning disability, physical illness etc), however given the high rate of ADHD among prisoners involved in institutional critical incidents, we need to lobby for ADHD to be prioritised. A substantial barrier to the identification of ADHD and the delivery of mental health care in prison is the high turnover of inmates. The prison population nears 90,000 with around 200,000 new names introduced each year, and over 50% of prisoners serve less than six months before moving on to community supervision. In addition, the frequency of inter-prison transfers means that data-sharing protocols across authorities will be essential.

### Forensic Mental Health Services

Rates of ADHD are disproportionately high in personality disorder wards in forensic mental health services (early data from an ongoing study at the high-secure Broadmoor Hospital indicate a prevalence of 25%), and addiction populations (20%) [25]. The persistence of ADHD symptoms has been associated with elevated rates of critical incidents (specifically verbal aggression and damage to property) within personality disordered patients detained under the Mental Health Act [17], and with the average length of stay in medium security being two to four years (and costing c.£170,000 per year) there is ample opportunity for a comprehensive screening and diagnostic programme to be introduced.

Within mental health services there is an existing infrastructure into which ADHD awareness will fit. In order to successfully build on this framework, two important factors were identified:

1) The development and provision of accessible information and resources for staff and patients and their families.

2) The development and provision of a monitoring checklist to record assessment and prescription information for the patient, which can be completed by multidisciplinary staff.

However, whilst routine screening is conducted on admission to forensic inpatient services, this is not routinely conducted in community services where the majority of ADHD offenders with mental disorder are likely to be found. Existing screening procedures, where provided, are unlikely to include ADHD, and in some cases ADHD may be misdiagnosed (e.g. as personality disorder), thus emphasising the importance of training for professionals in ADHD assessment and diagnosis, which does not currently feature in generic training curricula.

## Interventions for ADHD

The conclusion of NICE guidelines for the treatment and clinical management of adults with ADHD [1] was that ADHD needed to be screened for and recognised, following which a referral to an expert in the diagnosis and treatment of ADHD should be made. The recommended first line treatment for adults with ADHD is methylphenidate, followed by second line treatments with either atomoxetine or dexamphetamine. In high risk populations consideration should be given to the use of atomoxetine as the first line choice, where abuse and/or diversion of stimulant medication are considered potential risks. Drug treatments for ADHD should always be considered as part of a comprehensive treatment programme addressing psychological, behavioural and educational or occupational needs.

The treatment of ADHD in the prison population is expected to have three main benefits. First, the reduction of symptoms of ADHD that impact adversely on behaviour within the prison setting, such as inattentiveness, physical restlessness, impulsive responding and mood instability. Second, the reduction of ADHD symptoms will enable individuals within the prison system to take better advantage of rehabilitation programs aimed at the reduction of recidivism and improved behavioural control. Third, the treatment of underlying ADHD may lead to improvements in comorbid disorders such as antisocial and borderline personality disorders, substance abuse disorders including addiction, and anxiety and depression including the risk for suicide.

We can therefore see that treatment of ADHD within offender populations fits well with the Risk-Needs-Responsivity principle, which proposes that treatment is targeted at the riskiest cases and at needs relevant to the service (e.g. treatment targeting criminogenic needs in offending populations). Programmes that adhere to the Risk-Needs-Responsivity principle, with strong strategies for reducing criminality, have been shown to be particularly effective in rehabilitating offenders and reducing recidivism [26]. Working within this model, there are three broad aspects that relate to treatment for ADHD offenders:

1) Pharmacological treatments to alleviate ADHD symptoms.

2) Psychological treatments aimed at improving strategies for self-control and reduction of antisocial attitudes and behaviours.

3) Concurrent treatment of comorbid disorders.

Offenders with untreated ADHD can be particularly difficult to manage in prison/institutional environments. Individuals with high levels of ADHD symptoms were recently found to have an 8-fold greater number of critical incidents in a Scottish prison and a 6-fold greater number of critical incidents than inmates with Antisocial Personality Disorder [15]; mainly consisting of verbal and physical aggression. Critical incidents of this type have also been found in personality disordered patients screening positive for ADHD and who are detained under the Mental Health Act [17]. The Young study [15] further found that the increased rate of critical incidents among prison inmates with ADHD could not be accounted for solely by co-occurring behavioural disorders, since the association with ADHD remained significant after controlling for Antisocial Personality Disorder. This suggests that there is something about ADHD itself that leads directly to an increased rate of critical incidents with prison/institutional settings, and these behavioural problems might therefore be expected to respond to treatments that reduce levels of ADHD symptoms.

The reasons for the particularly high rates of behavioural disturbance with prison inmates with ADHD are likely to stem from several sources related to the core syndrome of ADHD, including impulsive responding, mood instability, emotional dysregulation and low frustration tolerance [27-30]. Gudjonsson and colleagues [31] also found that prison inmates with ADHD have a particularly chaotic or disorganised style of behaviour that may also contribute to their behavioural problems. However, we also know that ADHD is associated with the development of conduct disorder during childhood and adolescence and this may lead to antisocial behaviours in adulthood. ADHD is therefore an important risk factor for the development of later antisocial behaviour. Left untreated, ADHD is likely to be an exacerbating factor that maintains antisocial behaviour and reduces the ability of an individual to alter their behavioural patterns.

Clearly ADHD has a greater impact on people than just the core symptoms of the disorder. In most cases the disorder starts during early childhood and has a negative impact in many areas of life throughout the lifespan [reviewed in 1]. One view of ADHD, supported by available data, is that children with ADHD are particularly susceptible to risk factors for the development of behavioural disorders, such as background social environment and genetic factors, and the often adverse negative events resulting from ADHD such as poor social interactions, poor engagement with education and exclusion from mainstream activities. Thus treatment within criminal justice settings will usually require the integration of interventions for comorbid mental illness, personality disorder, substance misuse, psychological problems, educational and occupational needs, criminogenic and other offence related factors. Treatment of ADHD is expected to enhance the effectiveness of these important interventions by reducing key symptoms and behaviours that act as a barrier to recovery and rehabilitation; including greater control over emotional and impulsive responses, reduced levels of restlessness, increased ability to focus and plan ahead and improved ability to take part in psychological treatment programs.

### Pharmacological treatments for ADHD

The use of pharmacological treatments for ADHD in children is well established in the UK and across Europe, with approximately 1% of the child population receiving stimulants or atomoxetine for ADHD [32]. The pharmacological treatment of adults with ADHD is similar to that in children, since drug treatment trials have been found to be equally effective in adults as children [33]. Overall the effectiveness of stimulants or atomoxetine in adults compares well to other drug treatments for mental health disorders, such as the use of antidepressants to treat depression; and for this reason NICE [1] and other recent expert reviews [1,34] conclude that drug treatments for ADHD in adults are the first line choice when considering treatment options. This is particularly true when treating people with ADHD with severe levels of impairment and/or associated behavioural problems, when implementing rapid and effective treatments is thought be most important [1]. In adults there is as yet insufficient evidence to recommend psychological approaches as first line treatments, although this might be suitable in less severe cases. It is however important to pay attention to the NICE recommendation that drug treatments for ADHD should always be considered as part of a comprehensive treatment programme addressing psychological, behavioural and educational or occupational needs.

The recommended first line treatment for ADHD in adults is methylphenidate, followed by dexamphetamine or atomoxetine. Currently none of the drugs available to treat ADHD in the UK are licensed for use in adults, although treatment trials required by the regulatory bodies are underway that are expected to lead to extension of current licensing to the adult population. Atomoxetine is licensed for use in adults but only as a continuation of treatment first initiated during childhood or adolescence (before the age of 18 years). This situation is an anomaly because in many cases pharmacological treatments are licensed for use in adults but not paediatric populations and the risks associated with stimulants are not thought to be greater in adults. Particular concerns in adults include cardiovascular changes such as increased pulse and blood pressure that need to be carefully monitored, although this is similar to many other drugs used in adults. Despite these potential problems, having fully reviewed available evidence, UK national guidelines from NICE [1] recommend that in most cases pharmacological treatments are used once the diagnosis of ADHD has been made in adults.

The main treatment effects recorded in drug treatment trials are improvements in levels of inattention, hyperactive and impulsive behaviours and symptoms. Studies have also documented a wider range of improvements on social and academic function and an individual's overall sense of well-being. Some studies have specifically reported on reductions in aggressive behaviour, with stimulant effect sizes being similar to those reported for core ADHD symptoms [35]. An important series of studies investigated mood symptoms in addition to core ADHD symptoms and found similar effect sizes for both sets of symptoms when treating adults with ADHD with either stimulants or atomoxetine [27,28]. For example, in one study of methylphenidate it was found that there was a correlation in the improvement of mood symptoms with ADHD symptoms during the treatment process of around 0.8 [28].

The nature of the symptoms that improve with stimulant medication can best be understood from the descriptions given by patients being treated for ADHD [36]. The rapid onset and marked impact of stimulants on ADHD symptoms is widely reported by people with ADHD taking such treatments. Typically people say that within a short time of taking the medication they feel calmer, more focused and better able to initiate and complete tasks. They report improvements in their ability to focus their attention, greater motivation and reward from usual activities of daily life, improved ability to plan ahead with less forgetfulness and increased levels of self-organisation. Impulsive symptoms are reduced with less subjective and objective restlessness. Problems such as mood swings greatly reduce and they find that situations in which they were particularly prone to become irritable or aggressive, such as waiting turn in queues or being irritated by other peoples responses, are now far more easy to manage. Overall there is greater control over behaviour and people may find they can stop and think more easily, rather than acting in a more impulsive and unthinking way. Subjectively people find that their mind is much calmer, more relaxed and they are better able to focus their thoughts. This is often described as part of an overall reduction in both mental and physical overactivity. People with ADHD typically describe their mind as always on the go, a kind of ceaseless mental activity with multiple short lived or flitting thoughts going on at the same time. This kind of excessive and unfocused internal mental activity is often associated by people with the tendency to talk over or interrupt people or their difficulty in attending to what people are saying to them, including following simple instructions. Overall people treated for ADHD report numerous changes in their mental state and behaviour which can be best characterised as improved self-control over core processes such as attention, impulsive responding and emotional control.

### Delivery of drug treatments within the prison setting and abuse potential

Prescribing stimulant medication in CJS settings may be perceived as unattractive due to the drug being (currently) off-licence, the controlled drug status for stimulants and abuse potential. The potential for abuse was recognised by NICE who suggest that atomoxetine may be a better option where this is a particular concern because it is not a controlled drug and is a non-stimulant. However the overall effectiveness of stimulants, which NICE consider to be greater than atomoxetine, means that stimulants should also be considered either as a first line or second line choice. The delivery of medication within the prison setting should not however be a problem, since many prisons already run medication-based programmes for controlled drugs (e.g. methadone maintenance) and successfully adhere to protocols and policies that aim to reduce the chances of mismanagement.

The abuse potential for stimulants is however often overstated and usually by professionals who are not familiar with the effects of stimulants in the treatment of ADHD. First, we know from follow-up studies that the use of prescribed stimulants is not associated with an overall increase in drug abuse problems and may be associated with a reduction in illicit drug use [37-39]. Second, one of the main problems in treating children with ADHD as they grow older is keeping them on stimulant medication, even when this thought to be important to their continued mental health. This is because many adolescents no longer wish to engage in the treatment program and prefer to stop medication, even when it is perceived by others (parents, teacher or professionals) to be beneficial. There is therefore no indication that stimulants are addictive when prescribed for the treatment of ADHD. Third, studies in the US where stimulants are more widely prescribed point towards the main misuse of stimulants being diversion to increase performance at work or in education, however the rates of stimulant prescriptions in the US is far higher than in the UK to high functioning individuals where academic performance is the main concern. Overall the potential benefits of treatment, particularly in highly impaired individuals, appear to greatly outweigh the potential risks. Risk assessments should however be carried out in each individual case and consideration given to the particular drug formulations prescribed.

Drugs with low abuse potential include atomoxetine which is a non-stimulant and long acting formulation, those where the stimulant cannot be easily extracted for injection, such as methylphenidate OROS in the UK or skin patches and long acting lisdexamphetamine in the USA.

### Psychological treatments for ADHD

NICE recommends that drug treatment should always form part of a comprehensive treatment plan that includes psychological, behavioural and educational advice and interventions. Medication is likely to improve adherence to psychological treatments such as offender treatment programmes and other therapeutic, educational and occupational activities. Thus addressing ADHD may have a two-fold impact in crime reduction, first by directly treating the disorder (e.g. reducing symptoms) and secondly by improving engagement with rehabilitative programmes. Specific programmes have been developed that integrate the two, and there is some evidence from studies in children that psychological therapies, in combination with drug treatments lead to greater sustained effects and greater effects on comorbidity [40]. However, although recent research supports the use of cognitive behavioural methods for treating adult ADHD [41-43], treatment with psychological therapy remains an under-researched area and a priority for future research. Psychological and psycho-educational programmes are available that provide advice on how to adapt treatments to suit those with ADHD [e.g. 44, 45]. The R&R2 ADHD offender programme [45] for example, is currently being evaluated in a randomised controlled trial (RCT) in Iceland. Preliminary results from a community pilot study of R&R2 has shown it to be effective in treating ADHD adults with comorbid difficulties, with the effect continuing to improve at three-month follow-up [46].

### The commissioning of treatment

Providing access to regular treatments of the right kind is generally a commissioning matter, however the evidence base needs to be expanded to evaluate newly developed, specialist programmes. A useful starting point might be to simply promote awareness of ADHD among those facilitating treatments.

Treatment protocols in prison are supported by PCT commissioning through links to care standards in the wider community, and it may be beneficial to take a phased approach. It may be sensible to target those with longer sentences, maximising opportunity for initiation and optimisation of treatment. Identification and treatment of ADHD inmates is likely to reduce behavioural disturbance within the prison setting but additionally improve engagement with therapeutic, education and occupational activities. Education is provided on a smaller scale in prison than in the community (e.g. two or three to a class) and one-to-one attention will optimise motivation, co-operation and learning. Greater understanding about ADHD and associated problems will maximise treatment benefit and increase the chance of successful rehabilitation and constructive skills acquisition.

The NHS is now responsible for the delivery of prison healthcare, however in the past practitioners in forensic mental health services have lacked confidence in prescribing stimulants, perhaps due to a lack of clinical guidelines. Thus, treatment plans need to be multidisciplinary and comprehensive, and need to recommend stimulant/drug therapy as a precursor to psychological work addressing criminogenic factors.

In the short-term, outcome needs to be assessed using symptom screens and staff measures to assess behavioural improvement (e.g. in treatment engagement, reduction in institutional disturbance). Longer-term effects may include transfer to a lower (and therefore less costly) level of security with greater opportunity to access rehabilitation, and reduction in antisocial and criminal behaviour.

In the community, after discharge from prison, some individuals will have contact with probation staff and/or be subject to a Multi Agency Public Protection Arrangements (MAPPA) review. This service provides psychosocial support for prisoners in the community, thus effective links with local mental health services and support agencies, and information sharing is necessary.

## The Need for Integrated Pathways

**Figure 1:**
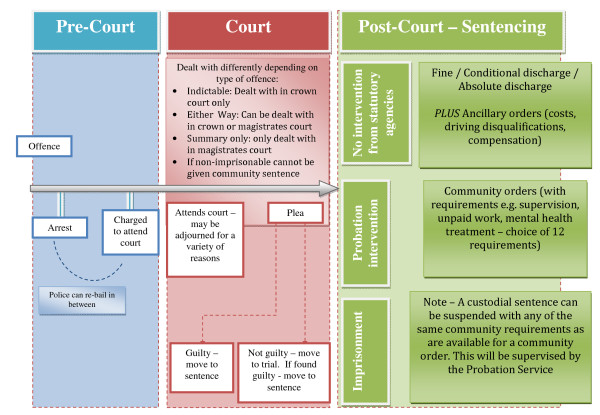
**Pathways through the Criminal Justice System**.

A common theme that arose during the meeting was the need for integrated care pathways between CJS agencies. Excellent service provision in one setting is of little benefit without continued care through integrated pathways. For persistent offenders, the pathway is not linear but often cyclical as they may move through stages multiple times (see Figure 1). It is crucial that continuity between services parallels the individual's progression though the system. Inevitably this will require effective IT systems and a new generation of systems will be delivered in 2010 providing improved links both between prisons and community care.

It was recognised that it is important to establish a continuous, integrated care pathway that follows the offender 'journey' from initial police contact through to eventual resettlement, and that interfaces health with the CJS services. This may include a criminal justice liaison to address factors that may impede justice or consider custodial alternatives for some individuals (e.g. community orders, treatment services). The contribution of mental health staff at court will improve identification of mental health issues, including ADHD. It will be important to develop joint (or comparable) risk and health assessments across CJS partners, and provide training and common information sharing protocols and management systems. Referral pathways post-identification must be effective with RCT research a priority, as a strong health economic case must be established.

The core NHS care within the CJS is provided by primary care services (GPs) and secondary psychiatric services, and the key to an integrated pathway for many offenders will be the transfer of care, especially for those leaving prison (e.g. via their GP). The GP is the gatekeeper for referrals to community services. For those offenders without a GP, PCTs aim to implement straightforward procedures to facilitate GP registration (some may not have been successful in the past due to communication barriers, inability to complete paperwork, etc). This process will be assisted by the probation service who are involved with offenders from before they leave prison in order to assess risk, and continue to mentor them in the community. This includes a multi-agency Reducing Reoffending Delivery Plan, which aims to reduce reoffending and ensures that all offenders have a GP. However, probation staff do not work with everybody leaving prison and those with short-term custody tariffs are unlikely to receive a probation service at all.

Awareness about ADHD and its implications (e.g. in different settings) throughout the whole care pathway will be essential in supporting ADHD offenders to rehabilitate into the community and make lasting change. This involves ensuring that services exist within the community to support offenders with ADHD in bringing about continuity of care. Gaining support from a keyworker or mentor will assist ADHD offenders to access continued care. The provision of psychoeducational materials about ADHD for voluntary sector community agencies and charities will assist them in their endeavours to support ADHD offenders in linking with healthcare, re-housing, and management of finances and employment.

However, we are in a climate of strong competition for resources; some individuals may require a lot of home supervision in the community, frequent medication monitoring/delivery and occupational support may also be required. One factor that will impact on service provision will be a move towards 'payment by results', which involves the clustering of detainees to correlate improvement over time with outcome measures. These clusters are likely to represent major sources of concern, such as schizophrenia. Adult ADHD patients may require the same amount of resources as severely psychotic patients but respond to treatment more quickly and effectively. This emphasises the need to develop an evidence base for the treatment of ADHD offenders and, critically, to include health economic modelling when evaluating outcome.

## Conclusions

The aim of putting health and social care at the heart of the CJS has governmental support. However, it is important that health and criminal justice agencies work together to find 'win-win' solutions for managing individuals and their care, in turn providing better justice, more efficient services and better health and social outcomes. At present, ADHD is not on the agenda, and bringing together key experts in the field proved difficult, with most being *either *experts in adult ADHD or experts within the CJS. This illustrates well the challenge that lies before us in raising awareness of adult ADHD within the CJS. Given the disproportionately high rates of ADHD offenders compared with the normal population and the association with violent, persistent offending, ADHD is a condition that the CJS cannot afford to ignore.

Training and work force development will be important to improve awareness and basic understanding of ADHD in order to signpost appropriate healthcare and rehabilitation. This needs to be introduced at every stage of the offender pathway to maximise the support that can be offered and the success of rehabilitation. Table 6 summarises key conclusions for screening, treatment, training, commissioning of ADHD forensic services and key areas of research. Whilst this consensus draws on the UK experience, with some adaptations it can be used as a template for similar local actions in other countries. Brief ADHD screening tools (with appropriate sensitivity and specificity) need to be introduced in all the criminal justice services as currently there is no such provision. Screening will alert a potential need and trigger a second stage process (e.g. diagnostic assessment, AA safeguard, an extension on Fast Delivery Reports to allow time for a more comprehensive probation report).

**Table 6:** Summary of key conclusions

**Screening and Assessment**
- Screening tools are needed in police custody suites, courts, prison and probation services; while screening procedures exist across CJS services with a range of sensitivities and specificities, these exclude ADHD.
- ADHD screening tools exist for this purpose (e.g. the Barkley ADHD scales [47] and the Adult ADHD Self-Report Scale [48] however initial screens may need to be briefer.
- For diagnosis, of particular importance is the issue of comorbidity, which can complicate symptom presentation and hinder identification of adult ADHD. Differentiating between diagnoses (e.g. between ADHD and personality disorder) requires distinct, evidence-based diagnostic tools with ADHD criteria specific to adulthood.
- Advice is available [49] and, as recognised by NICE, it will be necessary to include ADHD alongside other mental health conditions that currently have much greater awareness/training.
**Treatment**
- ADHD can be effectively treated by a range of therapies providing many opportunities and benefits of treatment across the CJS.
- Psychosocial interventions have been specifically designed for this (e.g. adapted R&R2 [45]).
- It may not be easy to encourage service managers and policy-makers to embrace new developments into care pathways and crime reduction strategies, but systems must be put in place for those with health needs who remain in prison care.
- Evidence for ADHD treatment is needed and must link with health economic modelling.
**Training**
- Little is known about the operational challenges of ADHD for prison staff and how these might be addressed.
- Greater understanding and awareness is key for improving assessment, diagnosis and treatment of adult ADHD, and continuity of care. This will require training across the CJS.
- Training in ADHD for medical staff is minimal, and needs to be increased.
- Training must extend beyond the medical discipline to all CJS agencies.
**Commissioning**
- PCT commissioning is the way forward in developing and modifying services, and a key issue in this regard is evidence.
- Establishing links between treatment and outcome is crucial (e.g. the direct correlation between methadone maintenance and reducing offending has demonstrated that detoxification programmes reduce both drug use and offending thus solving two problems).
- Both health and CJS commissioners will be attuned to interventions with the strongest evidence base. For ADHD this will require evidence of health and offence-related outcomes.
- Service provision is additionally determined by value for money, which further emphasises the need to demonstrate an economic argument for change within services.
**Key areas of research**
- Educational needs assessment across the CJS to determine knowledge, skills, attitudes and values, and identify training needs.
- Proof of principle studies to evaluate the use of screening measures across the CJS
- Proof of principle studies to evaluate treatment efficacy; using health and offence-related outcomes
- Cost-effectiveness studies using health economic modelling techniques to strengthen the case for ADHD treatment (e.g. each person prevented from entering prison saves £75,000p.a).

Having identified ADHD in an offender, appropriate support needs to be made available and the development of shared communication across disciplines/services via IT services will provide opportunities to help individuals who otherwise go without. GPs appear to be ideally placed for early identification and intervention, but variability in services due to the broad and complex range of individuals seen in primary care, and the different pathways through which individuals enter the CJS means this cannot be the only provision relied upon. Continuity of care is critical both through and after the CJS, with patient education and system training essential. It is important to bear in mind that, while there is a need for shared responsibility in terms of understanding and evaluating adult ADHD, services within the CJS are not surrogate health and educational services. Provisions need to be made in the community, in police stations, and in the transition from other services, to maximise the opportunity for identification, intervention and prevention.

A comprehensive research programme will be required to expand existing ADHD evidence into the realm of the CJS involving joint working and commissioning. In particular, proof of principle studies are needed to demonstrate effectiveness in health, behaviour and offence-related outcomes (including crime reduction). There are effective interventions for ADHD at any age, however a separate but related strategy for youth offenders will offer a preventative focus by providing crucial opportunities for treatment and support. It will be essential to include health economic modelling to demonstrate the service consumption costs of 'doing nothing' compared with the financial benefit of intervention and, potentially, prevention.

## Abbreviations

AA: Appropriate Adult; ADHD: Attention Deficit Hyperactivity Disorder; CJS: Criminal Justice System; DoH: Department of Health; DSM-IV: Diagnostic and Statistical Manual Fourth Edition; GP: General Practitioner; ICD-10: International Statistical Classification of Diseases Tenth Revision; MAPPA: Multi Agency Public Protection Arrangements; NHS: National Health Service; NICE: National Institute for Clinical Excellence; OASys: Offender Assessment System; PCT: Primary Care Trust; R&R: Reasoning and Rehabilitation; RCT: Randomised Controlled Trial; UKAAN: United Kingdom Adult ADHD Network.

## Competing interests

Support for the publication costs of this article was provided from an educational grant by Janssen-Cilag Ltd., Saunderton, Bucks HP14 4HJ, United Kingdom.

SY has been a consultant for Janssen-Cilag, Eli-Lilly and Shire; MA and UM for Janssen-Cilag; JT for Janssen-Cilag and Eli-Lilly; and PA for Janssen-Cilag, Eli-Lilly, Shire and Flynn Pharma.

SY has given educational talks at meetings sponsored by Janssen-Cilag, Shire and Flynn-Pharma, Novatis, Eli-Lilly; MA at meetings sponsored by Shire; UM at meetings sponsored by Bristol-Meyers Squibb, Eli-Lilly, Janssen-Cilag, Pharmacia Upjohn and UCB Pharma; JT at meetings sponsored by Janssen-Cilag and Eli-Lilly; and PA at meetings sponsored by Janssen-Cilag, Shire and Flynn-Pharma.

SY has received research grants from the National Institute of Health Research, Janssen-Cilag, Eli-Lilly and Shire; UM a research grant from Janssen-Cilag and grants from the Alexander von Humboldt Foundation, Medical Research Council (MRC) and Isaac Newton Trust; and PA has received a research grant from Shire, an educational grant from Janssen-Cilag and grants related to ADHD from Wellcome Trust, The Medical Research Council, US National Institute of Mental Health and the National Institute of Health Research.

SY and PA were members of the NICE guideline development group for ADHD.

MP and BB no disclosures at present.

## Authors' contributions

All authors contributed to the manuscript by discussing the issues at the expert UKAAN Forensic Special Interest Group Meeting held in London on 19^th ^and 20^th ^November 2009. The first draft manuscript was prepared by the lead author SY with contributions from all authors via electronic discussions and face-to-face meetings. A draft was reviewed by all the experts who attended the November meeting and other relevant colleagues (see acknowledgements section). The final manuscript was read and approved by all authors.

## Pre-publication history

The pre-publication history for this paper can be accessed here:

http://www.biomedcentral.com/1471-244X/11/32/prepub
